# Arginase and ceruloplasmin activity in the serum of patients with polysomnography-detected sleep bruxism

**DOI:** 10.3389/fneur.2025.1577869

**Published:** 2025-09-08

**Authors:** Helena Martynowicz, Monika Michałek, Marta Waliszewska-Prosół, Piotr Macek, Agnieszka Kusnerż, Gabriella Lachowicz, Jakub Przegrałek, Zuzanna Galińska, Rafał Poręba, Katarzyna Madziarska, Paweł Gać

**Affiliations:** ^1^Clinical Department of Diabetology, Hypertension and Internal Diseases, Wroclaw Medical University, Wrocław, Poland; ^2^Department of Neurology, Wroclaw Medical University, Wrocław, Poland; ^3^Department of Environmental Health, Occupational Medicine and Epidemiology, Wroclaw Medical University, Wrocław, Poland; ^4^Faculty of Medicine, Wroclaw Medical University, Wrocław, Poland; ^5^Department of Biological and Medical Foundations of Sport, Wroclaw University of Health and Sport Sciences, Wrocław, Poland

**Keywords:** sleep bruxism, ceruloplasmin, arginase, polysomnography, oxidative stress

## Abstract

**Background:**

Arginase and ceruloplasmin are enzymes of redox balance involved in the metabolism of nitric oxide. Arginase competes with nitric oxide synthase (NOS) for L-arginine and hence plays a crucial role in the arginase/NOS balance of maintaining proteins and the appropriate nitric oxide (NO•) level in the serum. On the other hand, ceruloplasmin (CP) is an acute- phase protein responsible for the metabolic balance of copper and iron. For this study it was to investigate the serum concentrations of enzymes involved in the redox balance, namely arginase type I (Arg1) and CP, in a group of patients with and without sleep bruxism (SB), which was diagnosed by polysomnographic examination.

**Methods:**

75 adults (35 women and 40 men, mean age 49.12) underwent a full- night of video polysomnography according to standards set out by the American Academy of Sleep Medicine. The concentration of Arg1 and CP in the serum, was determined using ELISA Kits.

**Results:**

The results showed that the concentration of Arg1 and CP was significantly lower in individuals with SB, irrespective of bruxism severity. Regression analysis revealed that only in the group of patients with higher Arg1 and CP concentrations, was there a negative linear relationship with the bruxism episode index (BEI).

**Conclusion:**

The results suggest that there is an oxidative imbalance in patients with SB, independent of the severity of bruxism. Higher plasma levels of Arg1 and CP were related to a lower BEI, potentially as the result of a protective biochemical balance against oxidative stress and inflammation in the SB.

## 1 Introduction

Arginase and ceruloplasmin are enzymes of redox balance involved in nitric oxide (NO) metabolism. Arginase is a manganese metalloenzyme involved in the urea cycle. This enzyme catalyzes the conversion of L-arginine to urea and L-ornithine (1, 2). Depending on the isoform, arginase participates in catalytic reactions involving L-arginine in various human tissues (3). Two arginase isoforms exist. Type I (Arg1) is found in the liver, while type II (Arg2) is expressed mostly in extrahepatic tissues, such as the kidneys, skin, endocrine glands and prostate (4, 5). Arginase competes with nitric oxide synthase (NOS) for the same substrate; thus, the arginase/NOS balance is crucial for maintaining endogenous concentrations of polyamines, proteins, and nitric oxide (NO•). Nitric oxide synthase (NOS) is an enzyme catalyzing the production of NO• in endothelial (eNOS), neuronal (nNOS), and immune tissues (inducible, iNOS). Endothelial nitric oxide synthase is a key enzyme in the production of the vasodilator, nitric oxide, which is an important component involved in appropriate vascular functioning (6). Arginase/NOS imbalance leads to disturbed homeostasis in the human body, influencing cell proliferation and fibrosis. Increased expression of arginase has previously been linked to harmful effects on the cardiovascular, immune and neurological systems of the human body. Previous studies have emphasized the role of arginase as a biomarker of disease development (7, 8).

Ceruloplasmin (CP) is an acute- phase protein and ferroxidase enzyme produced by the liver (9, 10). This multicopper oxidase plays a crucial role in the metabolic balance of copper (Cu) and iron (Fe) (11). It exhibits enzymatic functions such as ferroxidase, amine oxidase and catechol oxidase. Ceruloplasmin is involved in Cu transport, iron regulation (by oxidizing Fe2 + to Fe 3+), antioxidant processes (inhibits lipid peroxidation, promotes the synthesis of NO) and is implicated in minimalizing the deleterious effect of free radicals (scavenging). Furthermore, it also catalyzes the oxidation of a variety of substrates, such as Cu, Fe, and other organic substrates. While CP exhibits its oxidase function, it also plays a role in antioxidation (12, 13). The enzyme metabolizes NO in the plasma, ultimately producing nitrite and nitrosothiols (SNOs), which have been proposed to mediate protective responses to hypoxia and ischemia (14, 15).

Several neurodegenerative diseases such as Wilson’s disease, Alzheimer’s disease, and Parkinson’s disease are associated with disturbed Cu metabolism. However, the relationship between CP as an acute-phase protein with pro-inflammatory activity, and metabolic diseases, has also been demonstrated, including in its role in the development of diabetes, obesity, hyperlipidemia, and other cardiovascular diseases (16).

Sleep bruxism (SB) is defined as a multifactorial sleep-related movement behavior (17). Its role has been widely discussed in literature, highlighting both its harmful effect on oral health and/or its protective function (18). The working group focusing on this topic, has widely accepted the definition that SB is a rhythmic (phasic) or non-rhythmic (tonic) masticatory muscle activity occurring during sleep, and cannot be defined as either a movement or sleep disorder in otherwise healthy individuals (19). The potential etiology and pathomechanisms of SB have been and continue to be, discussed by numerous authors. Over time, extensive research studies have focused on the genetic basis of SB with the involvement of neurotransmitter receptors, increased anxiety and depression, stress perception, psychosocial state, exposure to substances (tobacco, alcohol, drugs, caffeine and environmental pollution) and certain comorbidities, such as obstructive sleep apnea (20) simple snoring (21) and periodic limb movement during sleep (22). Some authors have recently investigated the relationship between SB and inflammatory markers (23) however the body of evidence remains unclear. Michalek-Zrabkowska showed increased levels of CRP, and fibrinogen, probably as a result of stress and sympathetic activity in bruxers (24). The systematic review findings suggested that a higher intensity of SB could be associated with higher levels of proinflammatory parameters (25). Increased inflammatory markers are linked with increased cardiovascular risk. The inflammatory process is related to many disorders and is nowadays considered to be an adaptive regulation of homeostasis. Oxidative stress, expressed as an increased level of reactive chemical species like nitric oxide, is associated with inflammation and may lead to propagation of the inflammatory response (26).

Sleep bruxism has previously been linked to oxidative stress (27, 28) however data is limited and in these studies, sleep bruxism was diagnosed based only off of its clinical features, instead of by an objective polysomnographic examination (PSG).

Previously sleep bruxism has been linked to inflammation state and blood pressure variability (29). For this study, it was of interest to investigate the serum concentrations of enzymes involved both in the redox balance and nitric oxide pathway, namely arginase type I (Arg1) and ceruloplasmin (CP), in patients diagnosed with sleep bruxism by means of PSG examination.

## 2 Materials and methods

### 2.1 Participants

**Figure 1:**
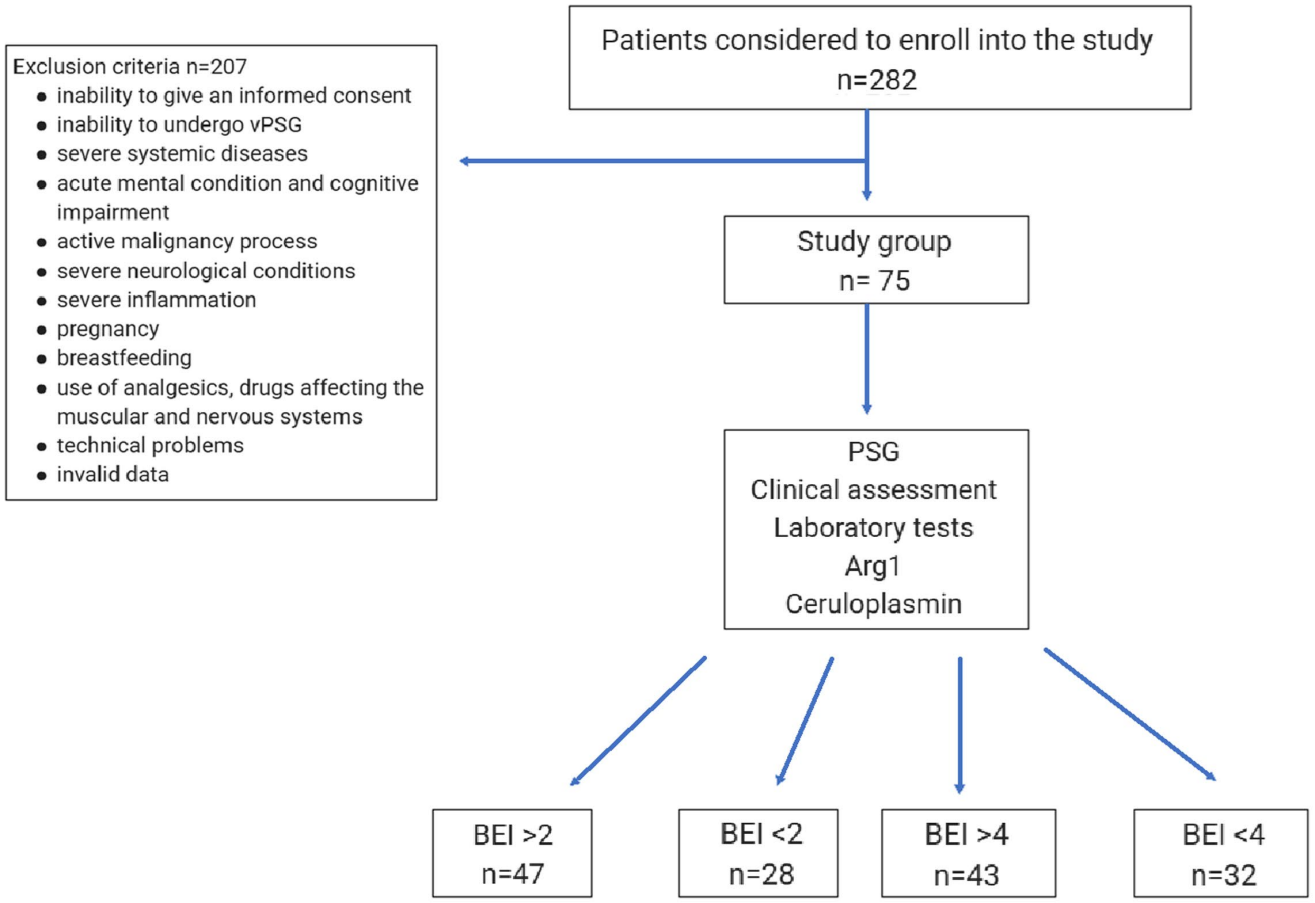
The recruitment process flowchart. PSG, polysomnography; Arg 1, arginase 1, BEI, bruxism episode index.

The clinical part of the study was conducted in the Sleep Laboratory at the Department of Internal Medicine, Occupational Diseases, Hypertension and Clinical Oncology at the Wroclaw Medical University in Poland. 75 adults were recruited for the study. Exclusion criteria were as follows: being unwilling or unable to provide informed consent to undergo polysomnographic examination, severe neurological conditions, history of treatment with medications affecting the functioning of the nervous and muscular systems, severe mental disorders and cognitive impairment, severe systemic diseases including active malignancy or an active inflammatory process, pregnancy and breastfeeding, addiction or use of analgesics and/or drugs affecting the muscles and breathing, neuropathic pain and severe rheumatic diseases. The study protocol is presented in Figure 1.

Participants of the study were assessed by a qualified physician and asked a series of questions about their comorbidities, weight and height, age, gender, currently used medications and stimulants.

### 2.2 Polysomnography

All patients who were qualified for the study, underwent a standardized single-night polysomnography examination, supplemented with audio and video recordings using the Nox-A1 (Nox Medical, Iceland) device. A certified physician assessed the polysomnograms (PSGs) according to guidelines set forth by the American Academy of Sleep Medicine (AASM), version 2.6. Polysomnograms were recorded between 22:00 and 06:00, to align with the natural circadian rhythm of the patients. The polysomnograms were analyzed in 30s epochs. The assessment included the evaluation of sleep latency, total sleep time and sleep efficiency (%), the extent of 1,2,3 non- REM sleep stages (N1, N2 and N3) and the rapid eye movement (REM) stage of sleep. Electrode placement adhered to the recommendations of the AASM (30). Respiratory events were detected by the nasal pressure airflow cannula. Apneic and hypopneic events were analyzed as follows: apneas were defined as a 90% cessation of airflow for ≥10 s, hypopneas were identified when the reduction in the breathing amplitude was by ≥30% for ≥10 s with a ≥ 3% decline in blood oxygen saturation, which was measured with a pulse oximetry device or was followed by an arousal. Saturation level (SpO2%) and pulse were recorded using a NONIN WristOx2 3,150 pulse oximeter (Nonin Medical Inc., Plymouth, MN, USA). The recordings were stored using Noxturnal software (Nox Medical, Reykjavík, Iceland).

Electromyographic (EMG) signals from bilateral masseter muscle activity during sleep, supplemented by audio and video recordings, were assessed and utilized to diagnose sleep bruxism events. These were then summated and expressed as the bruxism episode index (BEI). Events, including coughing, sleep- talking, yawning, snoring, swallowing of the saliva and face scratching, were excluded during the assessment of SB. Masticatory muscle activity events and bursts, assessed as bruxism episodes, were defined as being either phasic, tonic, or mixed, as per the AASM guidelines. A bruxism event was identified when an EMG amplitude peak was at least double that of the background EMG signal. When the interval duration between two EMG bursts was no longer than 3 s, the bursts were a part of the same SB episode. 2 s persistent events were classified as tonic SB, 3 or more bursts or “twitches” lasting over 2 s were qualified as phasic SB, while a combination of these elements was determined to be a mixed SB episode. The SB classification based on BEI is based on AASM guidelines and widely used in research on sleep bruxism. SB was categorized based on the frequency of bruxism episodes per hour of sleep (BEI) as non-SB (BEI < 2); SB (BEI > 2); or severe SB (BEI > 4) (30).

### 2.3 Laboratory tests

Blood samples were obtained from the participants by venipuncture at 7 a.m., after 12 h of overnight fasting, which were analyzed at the Laboratory of Wroclaw Medical University. Tests were conducted in accordance with the standard laboratory protocols of Wroclaw University Teaching Hospital. Venous blood samples were obtained into polyethylene terephthalate (PET) plastic tubes (Becton, Dickinson and Company; Franklin Lakes, NJ, USA), with K2EDTA. Blood samples were stored at − 70°C until subsequent analyses use.

The concentration of arginase 1 (Arg1) and ceruloplasmin in the serum was determined. Serum arginase 1 concentration was ascertained using the commercial test: Human Arginase-1 (Arg1) ELISA Kit (E4984Hu, BT Lab, Shanghai Korain Biotech, Shanghai, China), according to the manufacturer’s instruction. The detection range for this test is 0.5–200 ng/mL and the sensitivity 0.35 ng/mL. The coefficient of variation for intra-assay is 3.79–5.15% and for inter-assay <10%. Arg1 concentration is expressed in ng/mL.

Serum ceruloplasmin concentration was determined using the commercial test: Human Ceruloplasmin (CP) ELISA Kit (E0909h, Wuhan EIAab Science, Wuhan, Hubei, China), according to the manufacturer’s instructions. The detection range is 31.2–2000 ng/mL and the sensitivity 15 ng/mL. The coefficient of variation for intra-assay is 4.16–5.31% and for inter-assay 6.87–8.07%. Serum ceruloplasmin concentration is expressed in ng/mL.

### 2.4 Statistical analysis

Statistical analyses were conducted using Dell Statistica 13 software (Dell Inc., USA). The quantitative variables were expressed as arithmetic means and standard deviations, and the distribution of these variables was verified using the Shapiro–Wilk W-test. The qualitative variables were expressed as percentages. T test or the Mann–Whitney U-test was used for the evaluation of the independent quantitative variables in comparative analyses. Only the variables “REM (% of TST)” in polysomnography and “Potassium (mmol/L)” and “Calcium (mg/dL)” in laboratory tests were normally distributed. For these variables, the parametric *T* test was used in further analyses, for the remaining variables, the nonparametric U Mann–Whitney test was used. The relationships between the analyzed variables were determined by correlation and multivariate segmental linear regression with break-through point analyses. Results with *p* < 0.05 were statistically significant.

The study was accepted by the Ethics Committee of Wroclaw Medical University (no. KB-790/2022) and was conducted according to the guidelines of the Declaration of Helsinki. All the patients included had given written informed consent. The study was also registered in the international database for clinical studies- the Clinical Trials Database with the identifier NCT04937036.^1^

## 3 Results

The study group involved 75 Caucasian adult patients (35 women and 40 men, mean age 49.12 ± 16.91 years, age range 20–81 years). The demographic characteristics of the study group are presented in Table 1.

**Table 1:** Demographic characteristics of the study group.

Age (years)	*n* = 49.12
Men (%)	*n* = 40 (53.3)
Women (%)	*n* = 35 (46.7)
CAD (%)	*n* = 4 (6.15)
HT (%)	*n* = 27 (41.54)
DM (%)	*n* = 9 (13.85)
Stroke (%)	*n* = 3 (4.61)
CI (%)	*n* = 4 (6.15)
Tobacco (%)	*n* = 14 (21.86)

CAD, coronary artery disease; HT, hypertension; CI, cardiac infarction; DM, diabetes mellitus.

SB was diagnosed based on the polysomnographic evaluation. Mean polysomnographic parameters are presented in Table 2.

**Table 2:** Polysomnographic indices of the study group.

PSG parameter	Mean ± SD
AHI (*n*/h)	17.92 ± 19.24
ODI (*n*/h)	16.54 ± 17.75
Snore (% of TST)	22.18 ± 22.25
PLMS index (*n*/h)	9.20 ± 14.48
Sleep latency (min)	17.88 ± 20.93
REM latency (min)	105.89 ± 83.55
WASO (min)	69.16 ± 62.11
SE (% of TST)	81.3 ± 13.29
Mean SpO2 (%)	93.19 ± 2.43
Minimal SpO2 (%)	82.03 ± 9.35
Duration of SpO2 < 90% (% of TST)	10.33 ± 17.15
Average desat. Drop (%)	4.56 ± 2.30
N1 (% of TST)	6.98 ± 6.55
N2 (% of TST)	46.83 ± 19.33
N3 (% of TST)	29.63 ± 31.38
REM (% of TST)	22.06 ± 8.14
Arousals (*n*/h)	6.89 ± 5.54
Maximal HR (beats/min)	94.71 ± 10.07
Minimal HR (beats/min)	49.48 ± 21.71
BEI (*n*/h)	4.79 ± 4.36
Phasic SB (*n*/h)	2.49 ± 2.91
Tonic SB (*n*/h)	1.52 ± 1.55
Mixed SB (*n*/h)	0.78 ± 0.83
BEI supine (*n*/h)	8.37 ± 13.08
BEI non-supine (*n*/h)	3.22 ± 3.88
BEI in N1 (*n*/h)	19.00 ± 17.75
BEI in N2 (*n*/h)	05.05 ± 5.30
BEI in N3 (*n*/h)	1.95 ± 2.35
BEI in REM (*n*/h)	3.10 ± 03.05

BEI, bruxism episode index; AHI, apnea–hypopnea index; ODI, oxygen desaturation index; TST, total sleep time (min); SL, sleep latency; WASO, wake after sleep onset; SE, sleep efficiency; N1, sleep stage 1; N2, sleep stage 2; N3, sleep stage 3; REM, rapid eye movement sleep stage; mean SpO2, mean oxygen saturation (%); SpO2 < 90%, time with oxygen saturation <90% (% of TST); HR, heart rate; PLMS, periodic limb movements in sleep; SB, sleep bruxism.

**Figure 2:**
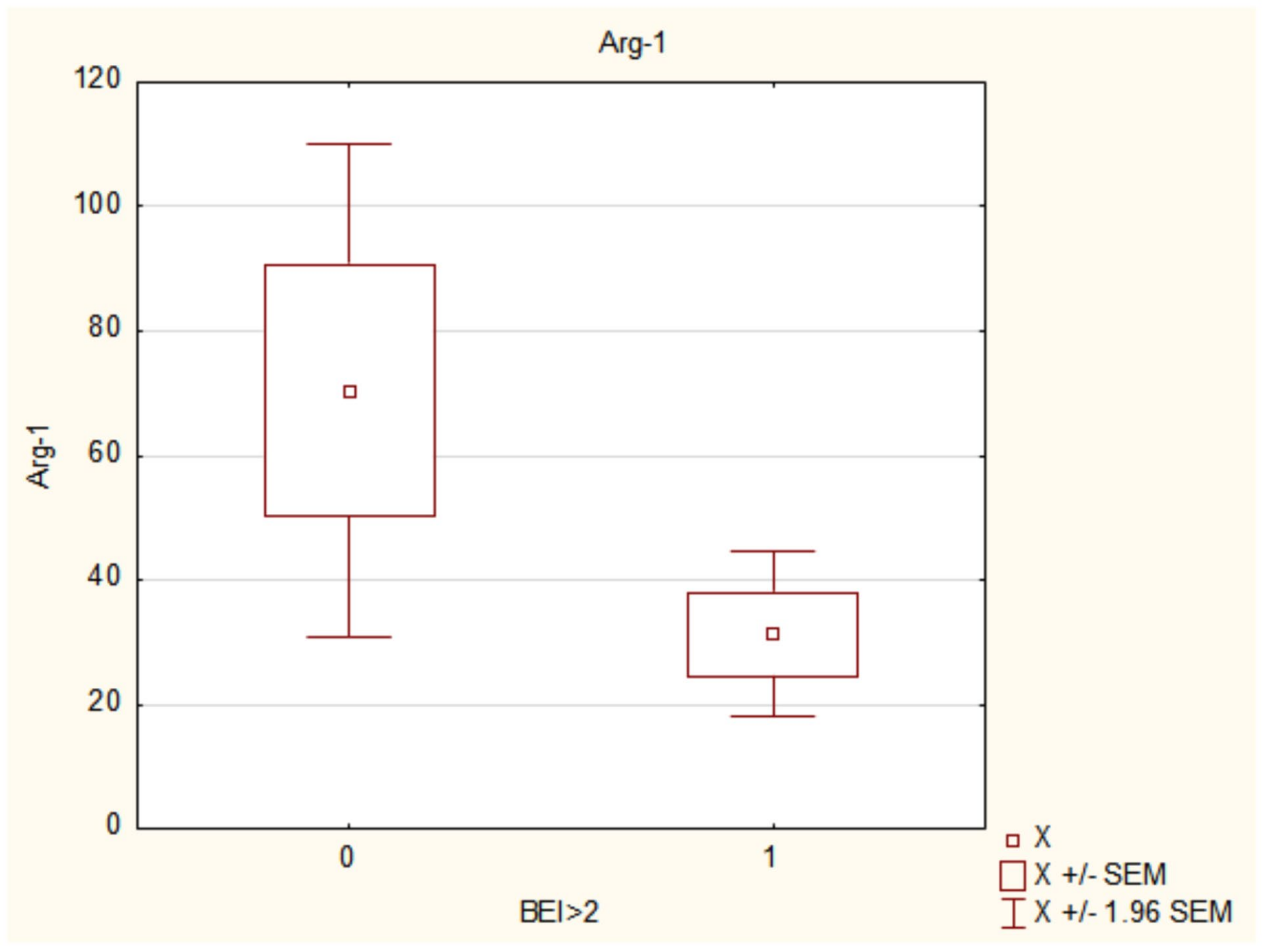
Arginase-1 (Arg-1) concentration in participants with diagnosed sleep bruxism (BEI ≤ 2 vs. BEI > 2). BEI, bruxism episode index; Arg1, arginase 1; SEM, standard error of the mean.

**Figure 3:**
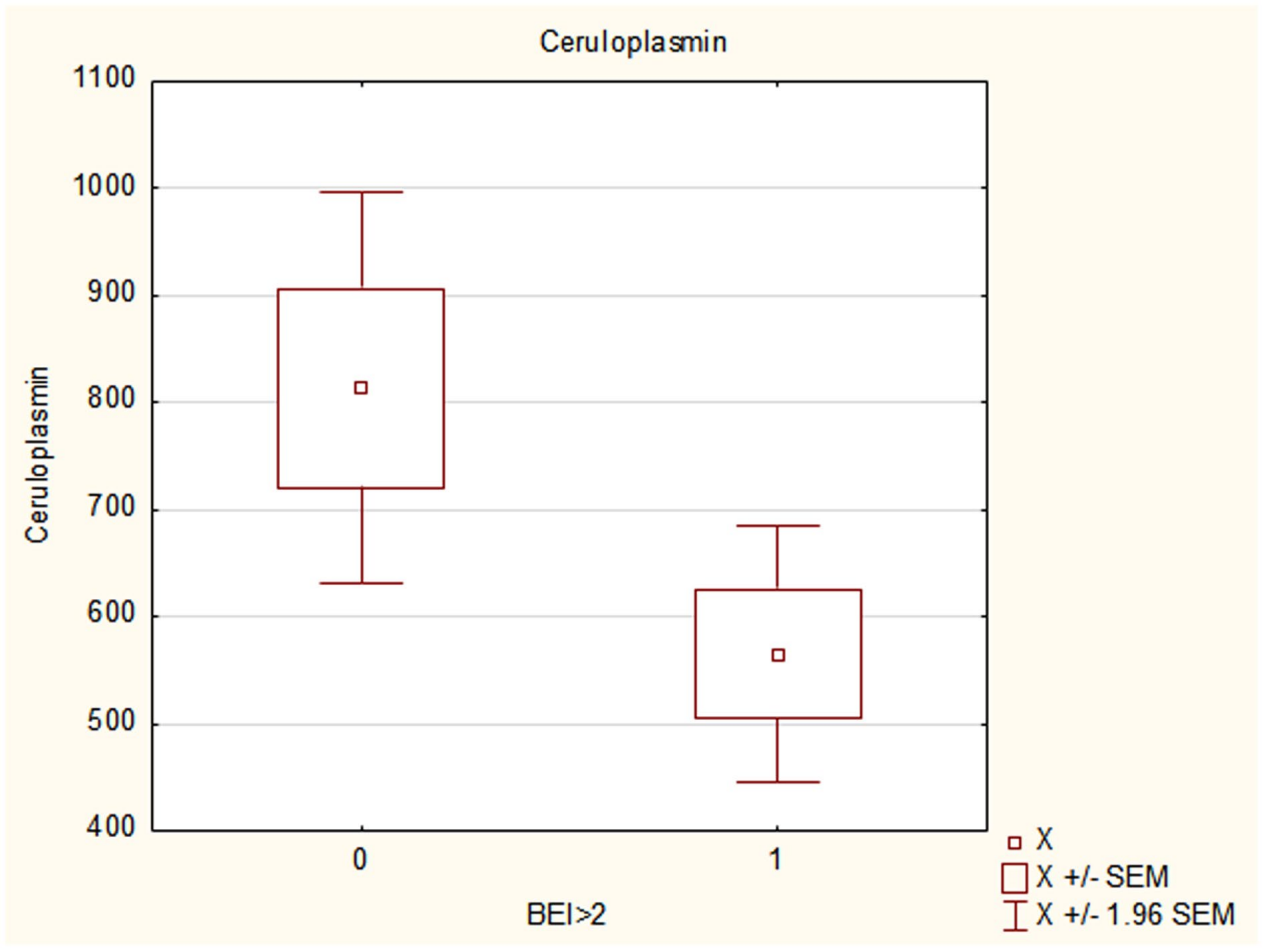
Ceruloplasmin concentration in participants with diagnosed sleep bruxism (BEI ≤ 2 vs. BEI > 2). BEI, bruxism episode index; SEM, standard error of the mean.

The results of this study showed that Arg1 and CP concentrations were significantly lower in individuals with SB, independent of bruxism severity. In the groups with higher BEI values, compared to the groups with lower BEI values (BEI > 2 vs. BEI < 2 and BEI > 4 vs. BEI < 4), the concentrations of Arg1 and ceruloplasmin were statistically significantly lower (see Figures 2, 3 and Tables 3, 4).

**Table 3:** Laboratory indices of the study group.

Laboratory parameter (number of samples)	Mean ± SD
Glucose (mg/dL) (*n* = 71)	24.78 ± 8.39
Creatinine (mg/dL) (*n* = 72)	0.94 ± 0.13
Uric Acid (mg/dL) (*n* = 67)	4.69 ± 1.15
Potassium (mmoL/L) (*n* = 71)	4.23 ± 0.39
Sodium (mmol/L) (*n* = 72)	140.65 ± 1.97
Calcium (mg/dL) (*n* = 60)	9.18 ± 0.22
Arg1 (ng/mL) (*n* = 74)	47.18 ± 79.41
Ceruloplasmin (ng/mL) (*n* = 71)	631.26 ± 433.13

Arg1, arginase 1; SD, standard deviation.

**Table 4:** Results for Arg1 and ceruloplasmin in bruxers and nonbruxers, differentiated based on bruxism severity.

Parameter	BEI<2 (*n*/h)	BEI>2 (*n*/h)	*p*	BEI<4 (*n*/h)	BEI>4 (*n*/h)	*p*
	Mean ± SD	Mean ± SD		Mean ± SD	Mean ± SD	
Arg1	77.04 ± 113.46	31.24 ± 47.24	**0.02**	68.37 ± 107.73	33.03 ± 49.03	**0.04**
Ceruloplasmin	739.08 ± 462.93	570.59 ± 413.71	**0.03**	718.48 ± 459.10	568.73 ± 413.77	**0.04**

BEI, bruxism episode index; Arg1, arginase 1; SD, standard deviation.

To determine the relationship between bruxism, Arg1 and ceruloplasmin, Spearman’s rank correlation analysis was subsequently performed (Table 5). Significant negative correlations were found between serum Arg1 concentration and BEI, tonic SB, mixed SB events and BEI in N1. No significant relationship was found between serum ceruloplasmin concentration and bruxism parameters.

**Table 5:** Bruxism indices correlated with Arg1 and ceruloplasmin (significant for *p* < 0.05).

Parameter	Arg1	Ceruloplasmin
BEI (*n*/h)	**−0.26**	−0.12
Phasic SB (*n*/h)	−0.21	−0.16
Tonic SB (*n*/h)	**−0.25**	−0.04
Mixed SB (*n*/h)	**−0.27**	−0.17
BEI supine (*n*/h)	−0.18	−0.18
BEI non-supine (*n*/h)	−0.23	−0.13
BEI in N1 (*n*/h)	−0.19	0.03
BEI in N2 (*n*/h)	**−0.24**	−0.12
BEI in N3 (*n*/h)	−0.17	−0.12
BEI in REM (*n*/h)	−0.22	−0.12

BEI, bruxism episode index; N1, sleep stage 1; N2, sleep stage 2; N3, sleep stage 3; REM, rapid eye movement sleep stage; SB, sleep bruxism; Arg1, arginase 1.

Since significant differences were found in both the comparative and correlation analyses, further relationships were explored.

Based on the multivariate segmental linear regression analysis with breakthrough point performed in the entire study group, the following relationship model for Arg1 was obtained:


Arg1=215.70–3.03BEIforArg1>127.88ng/mL(p<0.05)

and


Arg1=19.80–0.30BEIforArg1<127.27ng/mL(p>0.05)

The obtained model indicates that in the studied group, a higher BEI is a factor independently associated with lower serum Arg1 concentration, but only in the group of patients with serum Arg1 concentration >127.88 ng/mL. At lower serum Arg1 concentrations, the relationship between BEI and Arg1 becomes statistically insignificant.

The following relationship model for ceruloplasmin was obtained:


$$\begin{array}{l} \text{Ceruloplasmin}=1186.91–7.23\;\mathrm{BEI}\;\text{for ceruloplasmin}\\ >637.63\;\mathrm{ng}/\mathrm{mL}\;(p<0.05) \end{array}$$

and


$$\begin{array}{l} \text{Ceruloplasmin}=359.49–4.36\;\mathrm{BEI}\;\text{for ceruloplasmin}\\ <637.63\;\mathrm{ng}/\mathrm{mL}\;(p>0.05) \end{array}$$

The obtained model indicates that in the studied group, a higher BEI is a factor independently associated with lower serum ceruloplasmin concentration, but only in the group of patients with serum ceruloplasmin concentration >637.63 ng/mL. At lower serum ceruloplasmin concentrations, the relationship between BEI and ceruloplasmin becomes statistically insignificant.

Segmented linear regression analysis revealed that only in groups of patients with higher Arg1 (>127.88 ng/mL) and CP (>637.63 ng/mL) concentrations, significant negative linear relationships between Arg1 and BEI (*r* = −0.41, *p* < 0.05) and also between CP and BEI (*r* = −0.35, *p* < 0.05) were observed.

To evaluate differences between sexes, polysomnographic parameters were compared between female and male participants (Table 6). Bruxism parameters did not differ significantly between male and female participants.

**Table 6:** Polysomnographic indices in female and male subjects.

PSG parameter	Women	Men	*p*
	Mean ± SD	Mean ± SD	
AHI (*n*/h)	9.66 ± 16.61	25.15 ± 18.62	**0.00**
ODI (*n*/h)	8.95 ± 14.62	23.18 ± 17.74	**0.00**
Snore (% of TST)	12.4 ± 17.08	30.7 ± 22.93	**0.00**
PLMS index (*n*/h)	7.39 ± 10.49	10.78 ± 17.21	0.31
Sleep latency (min)	24.01 ± 27.66	12.51 ± 10.03	**0.02**
REM latency (min)	125.57 ± 96.09	88.68 ± 67.41	0.06
WASO (min)	73.41 ± 71.92	65.45 ± 52.73	0.58
SE (% of TST)	80.49 ± 15.29	82.01 ± 11.41	0.62
Mean SpO2 (%)	94.1 ± 2.22	92.4 ± 2.36	**0.00**
Minimal SpO2 (%)	84.51 ± 9.35	79.85 ± 8.91	**0.03**
Duration of SpO2 < 90% (% of TST)	5.92 ± 12.32	14.18 ± 19.83	**0.04**
Average desat. Drop (%)	4.05 ± 2.57	5 ± 1.97	0.07
N1 (% of TST)	6.03 ± 7.02	7.81 ± 6.08	0.25
N2 (% of TST)	49.07 ± 25.85	44.88 ± 10.88	0.35
N3 (% of TST)	34.44 ± 45.12	25.43 ± 7.44	0.22
REM (% of TST)	22.26 ± 8.23	21.88 ± 8.16	0.84
Arousals (n/h)	2.06 ± 10.5	0.1 ± 0.26	0.24
Maximal HR (beats/min)	95.14 ± 21.75	94.33 ± 21.95	0.87
Minimal HR (beats/min)	50.63 ± 12.58	48.48 ± 7.23	0.36
BEI (*n*/h)	4.73 ± 4.13	4.85 ± 4.62	0.91
Phasic SB (*n*/h)	2.38 ± 2.96	2.59 ± 2.9	0.75
Tonic SB (*n*/h)	1.58 ± 1.56	1.47 ± 1.56	0.77
Mixed SB (*n*/h)	0.78 ± 0.8	0.78 ± 0.87	0.99
BEI supine (*n*/h)	6.28 ± 6.63	10.25 ± 16.78	0.19
BEI non-supine (*n*/h)	3.19 ± 3.41	3.26 ± 4.33	0.94
BEI in N1 (*n*/h)	19.62 ± 18.93	18.44 ± 16.85	0.78
BEI in N2 (*n*/h)	5.03 ± 5.74	5.07 ± 4.95	0.98
BEI in N3 (*n*/h)	1.82 ± 2.2	2.06 ± 2.5	0.66
BEI in REM (*n*/h)	3.2 ± 2.59	3.02 ± 3.45	0.79

BEI, bruxism episode index; AHI, apnea–hypopnea index; ODI, oxygen desaturation index; TST, total sleep time (min); SL, sleep latency; WASO, wake after sleep onset; SE, sleep efficiency; N1, sleep stage 1; N2, sleep stage 2; N3, sleep stage 3; REM, rapid eye movement sleep stage; mean SpO2, mean oxygen saturation (%); SpO2 < 90%, time with oxygen saturation <90% (% of TST); HR, heart rate; PLMS, periodic limb movements in sleep; SB, sleep bruxism.

A similar analysis comparison was performed for age groups, with the median age of 49 years used to divide participants into younger and older subgroups (Table 7).

**Table 7:** Polysomnographic indices in different age groups.

PSG parameter	Older group (≥49 years)	Younger group (<49 years)	*p*
	Mean ± SD	Mean ± SD	
AHI (*n*/h)	6.35 ± 10.51	29.18 ± 19.19	**0.00**
ODI (*n*/h)	6.27 ± 9.32	26.53 ± 18.38	**0.00**
Snore (% of TST)	10.79 ± 17.74	33.28 ± 20.68	**0.00**
PLMS index (*n*/h)	5.43 ± 8.17	12.86 ± 18.07	**0.03**
Sleep latency (min)	13.06 ± 10.91	22.57 ± 26.72	**0.05**
REM latency (min)	119.71 ± 91.07	92.45 ± 74.28	0.16
WASO (min)	51.89 ± 66.85	85.98 ± 52.72	**0.02**
SE (% of TST)	86.12 ± 13.46	76.6 ± 11.44	**0.00**
Mean SpO2 (%)	94.64 ± 1.69	91.79 ± 2.23	**0.00**
Minimal SpO2 (%)	86.32 ± 6.49	77.84 ± 9.88	**0.00**
Duration of SpO2 < 90% (% of TST)	2.87 ± 7.4	17.59 ± 20.61	**0.00**
Average desat. Drop (%)	3.48 ± 0.89	5.6 ± 2.75	**0.00**
N1 (% of TST)	5.47 ± 6.78	8.45 ± 6.05	**0.05**
N2 (% of TST)	50.17 ± 24.97	43.64 ± 10.94	0.15
N3 (% of TST)	32.64 ± 43.86	26.7 ± 9.23	0.42
REM (% of TST)	22.91 ± 7.47	21.19 ± 8.75	0.35
Arousals (*n*/h)	0.22 ± 0.66	1.79 ± 10.14	0.35
Maximal HR (beats/min)	100.42 ± 18.56	89.13 ± 23.34	**0.02**
Minimal HR (beats/min)	50.95 ± 6.4	48.08 ± 12.61	0.22
BEI (*n*/h)	4.86 ± 4.07	4.73 ± 4.69	0.90
Phasic SB (*n*/h)	2.8 ± 3.11	2.18 ± 2.71	0.37
Tonic SB (*n*/h)	1.28 ± 1.43	1.77 ± 1.65	0.17
Mixed SB (*n*/h)	0.79 ± 0.77	0.76 ± 0.89	0.87
BEI supine (*n*/h)	5.93 ± 5.5	10.81 ± 17.45	0.11
BEI non-supine (*n*/h)	3.59 ± 3.85	2.9 ± 3.93	0.46
BEI in N1 (*n*/h)	20.76 ± 18.82	17.24 ± 16.67	0.40
BEI in N2 (*n*/h)	5.07 ± 5.58	5.02 ± 5.09	0.97
BEI in N3 (*n*/h)	2.31 ± 2.83	1.58 ± 1.71	0.18
BEI in REM (*n*/h)	3.25 ± 3.2	2.96 ± 2.94	0.69

BEI, bruxism episode index; AHI, apnea–hypopnea index; ODI, oxygen desaturation index; TST, total sleep time (min); SL, sleep latency; WASO, wake after sleep onset; SE, sleep efficiency; N1, sleep stage 1; N2, sleep stage 2; N3, sleep stage 3; REM, rapid eye movement sleep stage; mean SpO2, mean oxygen saturation (%); SpO2 < 90%, time with oxygen saturation <90% (% of TST); HR, heart rate; PLMS, periodic limb movements in sleep; SB, sleep bruxism.

## 4 Discussion

Oxidative stress and redox balance represent key topics in the investigation of the pathophysiology of cardiovascular disorders and other conditions affecting the human body. Oxidative stress is defined as an imbalance between the generation of reactive oxygen species, and the level of antioxidants. This field is maturing, as advanced *in vitro* and *in vivo* methods are continuously being developed. Redox homeostasis is crucial for a whole range of enzymatic processes taking place in the intracellular and extracellular space.

Arginase is a crucial enzyme involved in the urea cycle and plays a role in maintaining endogenous concentrations of proteins and nitric oxide. An increased expression of arginase is also considered to be a biomarker of cardiovascular, neurological and neoplastic disease development. Ceruloplasmin, a protein involved in serum copper transport, plays a protective role against oxygen radicals generated during oxidative reactions. A comprehensive understanding of the CP role in copper metabolism, lipoprotein oxidation and atherosclerosis development, in other words cardiovascular and metabolic diseases, constitutes an important area of research (16).

Some authors emphasize a potential link between sleep bruxism and increased cardiovascular risk. As is well known, one of the most crucial cardiovascular risk factors is systemic inflammation. Hence, previous studies were performed to solve this problem. For instance, the following studies were conducted on this topic (23, 24, 31), and concluded that biochemical parameters of inflammation were linked with a higher number of SB events. Nevertheless, potential biases persist in these studies, and several questions remain unresolved.

As far as we know, no previous research has investigated the serum concentration of Arg1 and CP, enzymes of redox balance involved in nitric oxide pathway, in sleep bruxers.

Sleep bruxism has been considered as a simple oral pathology associated with tooth wear and damage and jaw pain over a decade (32). However, since studies showed the central origin of the disorder, a growing interest in systemic implication of SB has been observed. Indeed, studies showed associations between SB and blood pressure (29, 33, 34), systemic inflammation (24, 31), hormonal disturbances (35), and sleep architecture (36). Recent studies indicate new pathomechanisms of blood pressure variability in sleep bruxers (33, 37). Worth noting, nitric oxide plays a crucial role in regulating blood pressure. It acts as a vasodilator improving endothelium function and as a result decreasing blood pressure. Both enzymes studied, Arg-1 and CP are involved in NO pathway. Thus, we aimed to assess these enzymes in sleep bruxism.

Arg1 and CP play also a role in the oxidative balance of the body, thus the serum concentration of these enzymes in SB patients was determined in this study, to illuminate a rather unchartered territory. Seminal contributions have been made by Kara et al. (27) and Ozcan-Kucuk et al. (28), who measured the total oxidant and antioxidant status in the plasma of bruxers. However, a major concern regarding the aforementioned findings is that the diagnosis of sleep bruxism was based on clinical criteria and patient history, rather than on objective assessment methods such as polysomnography. Recently, the relationship between sleep bruxism and redox balance parameters (total antioxidant status, advanced protein products and thiobarbituric acid-reacting substances) has been shown (38).

The results of this study demonstrated that Arg1 and CP concentrations in the serum were significantly lower in individuals with SB, independent of the severity of bruxism. This result highlights the point that little is known about the mechanisms linked with the pathophysiology of sleep bruxism. Contrary to the previous findings on the possible relationship between SB and SCI, systemic chronic inflammation (25), as well as to studies investigating oxidative stress in bruxers (27, 28, 38), current findings suggest that markers of impaired redox balance and proinflammatory features were lower in the SB group. Although Arg1 and CP are associated with several pathological processes, e.g., increased cardiovascular risk, immune-mediated reactions, and atherosclerosis, the results show that these enzymes do not seem to be associated with the severity of bruxism. It is difficult to explain such results within the context of discussion on “whether sleep bruxism contributed to the oxidative imbalance or whether oxidative imbalance contributed to sleep bruxism” (28). Considering these concerns, the results of the current study reflect the oxidative imbalance in the group of patients with sleep bruxism. Several reactive oxygen/nitrogen species can be transformed in numerous independent mechanisms involving enzymatic cascades, thus the implications of the current research should be replicated in future studies.

Regression analysis revealed that only in the group of patients with higher Arg1 and CP concentrations, does a negative linear relationship with BEI exist. These results suggest that impaired oxidative stress in bruxers could promote certain mechanisms involved in Arg1 and CP activity. As previously mentioned, Arg1 is a marker of cardiovascular impairment and disease development. Based on the results obtained, it can be speculated that decreased Arg1 and CP levels in the SB group may reflect a protective role of these enzymes in individuals with sleep bruxism. This is particularly relevant given that sleep bruxism has previously been associated with cardiovascular implications (39). However, reduced systemic antioxidant capacity should be also considered.

The association between sleep bruxism and cardiovascular risk is significant given the high prevalence of sleep bruxism in Western societies. The mechanisms linking sleep bruxism and cardiovascular disease are poorly understood and include endothelial dysfunction, pro-inflammatory state, and oxidative stress caused by sleep alternation, REM-non-REM balance, and sleep fragmentation (40). The latter, in particular, has been linked to sympathetic overdrive and cardiovascular risk. However, further research is needed to elucidate the complex mechanisms of cardiovascular changes in individuals with sleep bruxism.

The approach utilized in the study suffers from the limitation of a cross-sectional design, lack of an adaptive night before conducting PSG examination and an increased risk of bias due to the heterogeneity of the participants. Moreover, no proinflammatory markers nor redox markers were estimated. On the other hand, a relatively large study group as well as the ability to conduct polysomnographic evaluation of bruxism events, has proven to be beneficial in this field. To the best of our knowledge, this is the first study exploring the relationship between serum arginase 1 and ceruloplasmin concentrations in sleep bruxers vs. adults without SB.

Nevertheless, understanding the pathophysiological basis of SB in the context of oxidative stress should be explored in future work.

## 5 Conclusion

The findings of the study showed decreased levels of arginase 1 and ceruloplasmin, suggesting an association between tooth grinding and enzymes involved in the nitric oxide pathway, irrespective of bruxism severity. The results can indicate redox imbalance and possible cardiovascular risk in sleep bruxism. One potential issue in interpreting these findings lies in the nonspecific nature of arginase 1 and ceruloplasmin as markers. Their concentrations may be influenced by various systemic or local factors unrelated to bruxism, which could introduce uncertainty in the biological interpretation of the results. The main limitations of the present study are its cross-sectional design and the lack of direct assessment of proinflammatory and redox markers. Given that arginase 1 and ceruloplasmin levels may indirectly reflect redox imbalance, future research should adopt a more direct investigative approach. Further studies focusing on the nitric oxide pathway in sleep bruxism are warranted to better elucidate these complex interactions.

## Data Availability

The raw data supporting the conclusions of this article will be made available by the authors, without undue reservation.

## Footnotes

^1^ www.ClinicalTrials.gov
